# Steerable Delivery Sheath for Left Atrial Appendage Closure in Patients With Severely Enlarged Left Atria

**DOI:** 10.1016/j.jscai.2023.101290

**Published:** 2024-03-22

**Authors:** Nicolas Amabile, Ayoub Belfekih, Vincent Balmette, Khalil Mahmoudi, Nicolas Mignot, Clemence Roig

**Affiliations:** a Cardiology Department, Institut Mutualiste Montsouris, Paris, France; b Institut Cardiovasculaire Paris Sud, Massy, France; c Electrophysiology Department, Institut Mutualiste Montsouris, Rythmopôle, Paris, France

**Keywords:** atrial fibrillation, left atrial appendage closure, left atrium remodeling

## Abstract

**Background:**

Enlarged left atrium (LA) is frequently identified in patients who undergo left atrial appendage closure (LAAC) and negatively affects the device’s final position and intervention results. Steerable delivery sheath (SDS) could represent an option to overcome these difficulties. We aimed to assess the feasibility of SDS-assisted procedures and compare their efficacy to the standard sheath strategy.

**Methods:**

This study analyzed patients from our institution’s LAAC registry who had severe LA enlargement on CT scan (index LA volume >90 mL/m^2^) and underwent AMULET device implantation between January 2019 and March 2023. The patients underwent postprocedural CT scan to assess the device’s position, residual LAA filling, and peridevice leaks.

**Results:**

A total of 195 consecutive patients were screened and 47 (24%) met the inclusion criteria (n = 22 SDS group; n = 25 standard group). There was no difference in baseline clinical, anatomical, or procedural characteristics. The procedural technical success (96%) and complication rates (9% vs 4%; *P* = .59) were comparable in both groups. Post-LAAC CT scans were obtained in 19 SDS group and 22 standard group patients. We observed significantly lower incidences of residual patent LAA (26% vs 72%; *P* = .005), peridevice leaks (16% vs 64%; *P* = .004), and off-axis device position (0% vs 27%; *P* = .02) in SDS compared to the standard group, suggesting a better LAA sealing.

**Conclusions:**

Severe LA enlargement was frequent among LAAC candidates. In this situation, the use of SDS appears feasible and safe, leading to more efficient closures on follow-up imaging without a higher risk of periprocedural complications.

## Central Illustration

**Figure:**
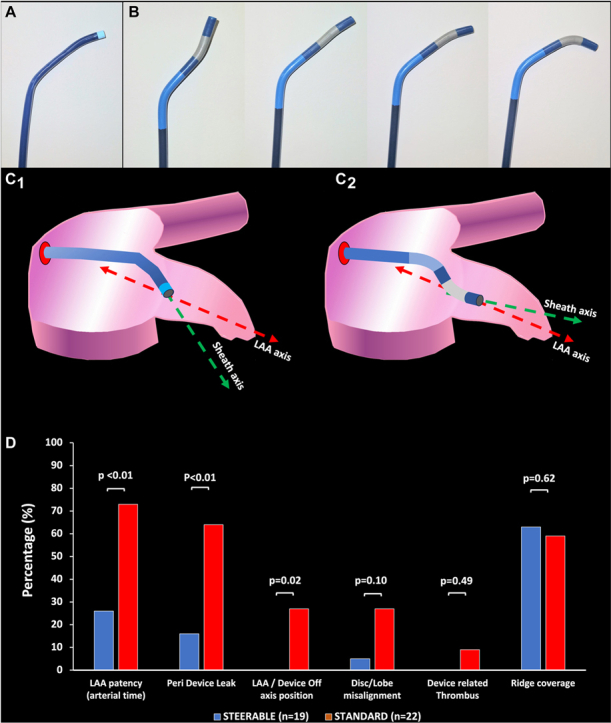

## Introduction

Percutaneous left atrial appendage closure (LAAC) has emerged as a valid option for the prevention of thromboembolic events in patients with nonvalvular atrial fibrillation (AF) and contraindications for oral anticoagulation.^1^ However, despite the improvement in the device’s design, the procedure's success may be limited by the high variability and complexity of left atrial appendage (LAA) morphology. Hence, prosthesis implantation might be impacted by the LAA angulation, orientation, or dimensions of the left atrium (LA) in case of complex anatomies, in which case a correct alignment of the prosthesis with the appendage can hardly be obtained by the use of conventional fixed-curves delivery sheaths.^2^ In these latter cases, the use of a flexible steerable delivery sheath (SDS) might represent a potential option to obtain more predictable and efficient results.^2^^,^^3^

LA enlargement is a frequent finding among patients with AF and is defined as index LA volume >32 mL/m^2^ by echocardiography and >40 mL/m^2^ by CT scan (significant LA enlargement: index LA volume >60 mL/m^2^ by echocardiography and >70 mL/m^2^ by a CT scan).^4^, ^5^, ^6^ Severe LA enlargement can induce difficulties in optimally visualizing the LAA by perprocedural transesophageal echocardiography (TEE), which affects the procedure guidance and quality. In addition, LA enlargement distorts angles and distances between the interatrial septum and LAA ostium, leading to nonoptimal device deployment within the appendage. Thus, enlarged LA has been reported to be associated with higher risk of patent LAA on CT scans after LAAC.^7^

The aim of the present pilot study was to compare the procedural and postprocedural results of SDS and standard sheath-assisted LAAC interventions in patients with severe LA enlargement.

## Methods

### Patient selection

This single-center study analyzed AF patients treated with percutaneous LAAC enrolled in our institution (Institut Mutualiste Montsouris, Paris, France) between January 2019 and March 2023. These patients were part of our prospective institutional registry for which they provided informed consent before inclusion. The patients were indicated for LAAC patients in case of the following: (1) paroxysmal or persistent/permanent nonvalvular AF with high embolic risk; (2) formal and definitive contraindication to oral anticoagulation therapy or recurrent ischemic event in patients under oral anticoagulation therapy.

The current analysis inclusion criteria were as follows: (1) LAAC procedure with an AMULET device (Abbott) implantation; (2) severe enlarged LA on preprocedural CT scans (defined as index LA volume >90 mL/m^2^, see below). Exclusion criteria were as follows: severe renal failure (creatine clearance <20 mL/min) with contraindication to CT scan, inability to consent, and LAA too small or too large for percutaneous closure with an AMULET device.

For each patient, the medical history, demographics, comorbidities, clinical and laboratory data, and CT scan characteristics were recorded prospectively by patient interview and medical record review.

The SDS group included all the patients who underwent LAAC with the steerable sheath between August 2021 and March 2023. The patients who underwent intervention with the conventional sheath between January 2019 and July 2021 were included in the standard control group. The patients who benefited from LAAC before January 2019 in our institution (including n = 92 cases of AMULET device implantation) were excluded from the analysis in order to minimize the potential bias related to any learning curve effect. The current study compared the data recorded obtained from the SDS group to the same clinical and CT scan data retrospectively analyzed in the standard group.

The study was approved by the relevant local ethics committee and performed in accordance with the Declaration of Helsinki and its amendments.

### SDS vs standard sheath

The Amplatzer SDS combines a fixed 45° proximal curve and a distal steerable curve that allows tip flexibility and may be oriented from 0° to 120° thanks to a bidirectional articulation (Central Illustration).^2^ The sheath can also be classically rotated clockwise or counterclockwise. ^2^ The addition of both mechanisms optimizes the coaxialty with the LAA axis and allows improved alignment between the device and appendage axes (Central Illustration). The sheath's inner diameter and outer diameter are 14F and 19F respectively. On the opposite, the standard TorqueVue (Abbott) sheath has two 45° fixed curves on its distal portion and an outer diameter ranging between 12F and 14F according to the models (inner diameter: 10F-12F) and can only be advanced or rotated within the appendage (Central Illustration).

**Central Illustration:**
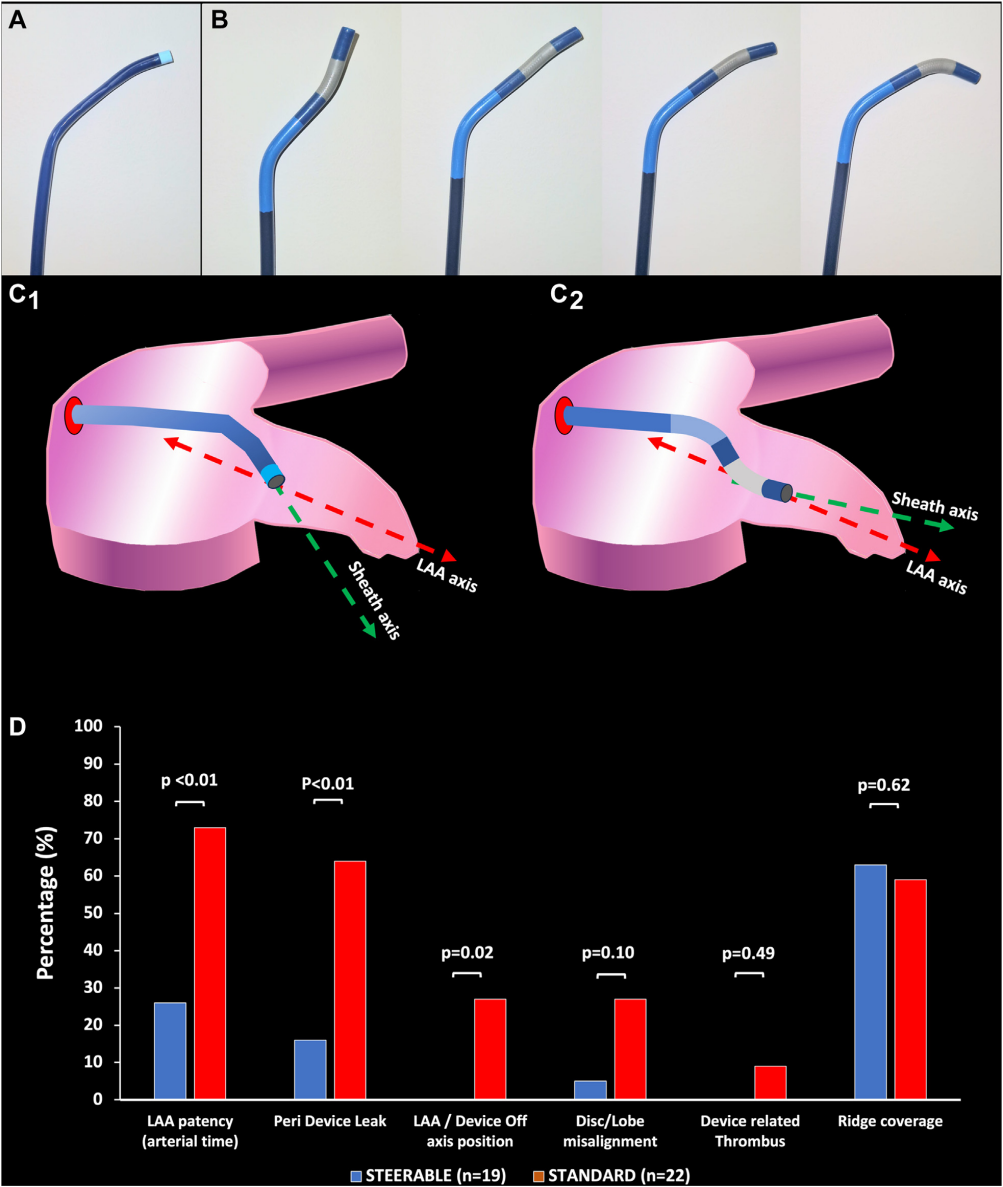
(**A**) The TorqueVue fixed-angle delivery sheath. (**B**) The Amplatzer steerable delivery sheath with multiple tip deflections. (**C**) Differences in coaxial alignment between left atrial appendage (LAA) axis and standard (C1) or steerable (C2) delivery sheaths. (**D**) Differences in LAA closure and device position on follow-up imaging between steerable delivery sheath and standard patients.

### LAAC procedure

All patients underwent AMULET implantation according to the latest consensus^8^ and using the standard or steerable delivery sheath.^2^ The procedures have been extensively described elsewhere.^9^ Briefly, the right femoral vein was punctured under ultrasound guidance. A vascular preclosing system (Proglide, Abbott) was used before inserting the transeptal puncture sheath. In case the SDS (Amplatzer Steerable Delivery Sheath, Abbott) was used, we implanted 2 preclosing devices with 30° to 45° rotation as previously described in transaortic catheter aortic valve replacement, because of the large outer catheter diameter and the theoretical risk of vascular injury.^10^ The transseptal puncture was performed through the right femoral vein access aiming the infero-posterior or infero-mid zone and the left upper pulmonary vein was then catheterized or a pig tail shaped stiff exchange wire (Safari, Boston Scientific) was inserted in the LA. The delivery sheath was inserted into the LA and advanced subsequently into the LAA. In case the SDS was used, a continuous flush of the internal lumen was provided by an external saline infusion line to prevent air embolism according to the manufacturer’s instructions for use.

The LAA landing zone (LZ) (as identified on preintervention CT scans) was measured by 2D and real-time 3D TEE methods,^6^^,^^11^ leading to the final choice of prosthesis size based on the manufacturer’s recommendations. The device was placed in the appropriate position using a combination of TEE and fluoroscopy, as recommended. The quality of the deployment (including device compression and stability) and the existence of potential leaks were controlled by 2D and 3D TEE. A compression rate >10% in all sections of the device on TEE was mandatory to proceed to the final release of the prosthesis. In case this was not obtained, the device was repositioned in a different position or changed for a larger model. The postimplantation antithrombotic therapy was left at the discretion of the operators according to the indication for LAAC and baseline clinical characteristics.

### CT scan procedures and analysis

Both preimplantation and postimplantation CT scans were performed according to the same protocol, by using either a prospective high-pitch flash mode or broad coverage single shot/step and shoot ECG-gated CT acquisition technique typically at the end-systolic phase. Higher tube voltages were selected to mitigate the potential artifacts related to the presence of the metallic device. Images were reconstructed using iterative reconstruction or filtered back-projection at 0.75 mm slice width, and 0.5 mm slice increment. The preimplantation and postimplantation CT scan images were centrally analyzed by 2 operators not involved in the procedure and not aware of the procedural strategy by using a 3D reconstruction dedicated software (3mensio, Pie Medical) according to the latest consensus.^6^^,^^12^ In the event of inconsistent adjudication, a consensus between all of them was required. The detailed methodology for CT analysis has been extensively described elsewhere.^13^^,^^14^

#### Preprocedural CT scan

The LA volume was measured at the end of ventricular systole when the LA was at its maximum size. Planimetration of the entire LA was performed in the transversal view using 12 to 15 slices.^15^^,^^16^ Volume was calculated using Simpson's method by multiplying the area of each manually traced LA (excluding pulmonary veins and LAA) by the section thickness and summing the volumes of the separate sections.^15^^,^^16^ The LA volume was indexed to body surface area. Severe enlargement was defined as index LA volume >90 mL/m^2^.

The LAA morphology classification (cauliflower, chicken wing, cactus, windsock) was based on the number of lobes and appendage angulation according to the consensus document.^12^ The LAA LZ was defined as the cross-section of the appendage that was perpendicular to its axis and connected the circumflex artery to a point 1 to 2 cm inside the LAA.^17^ The appendage length was the distance between the LZ and the tip of the LAA.

#### Postprocedural CT scans

**Figure 1:**
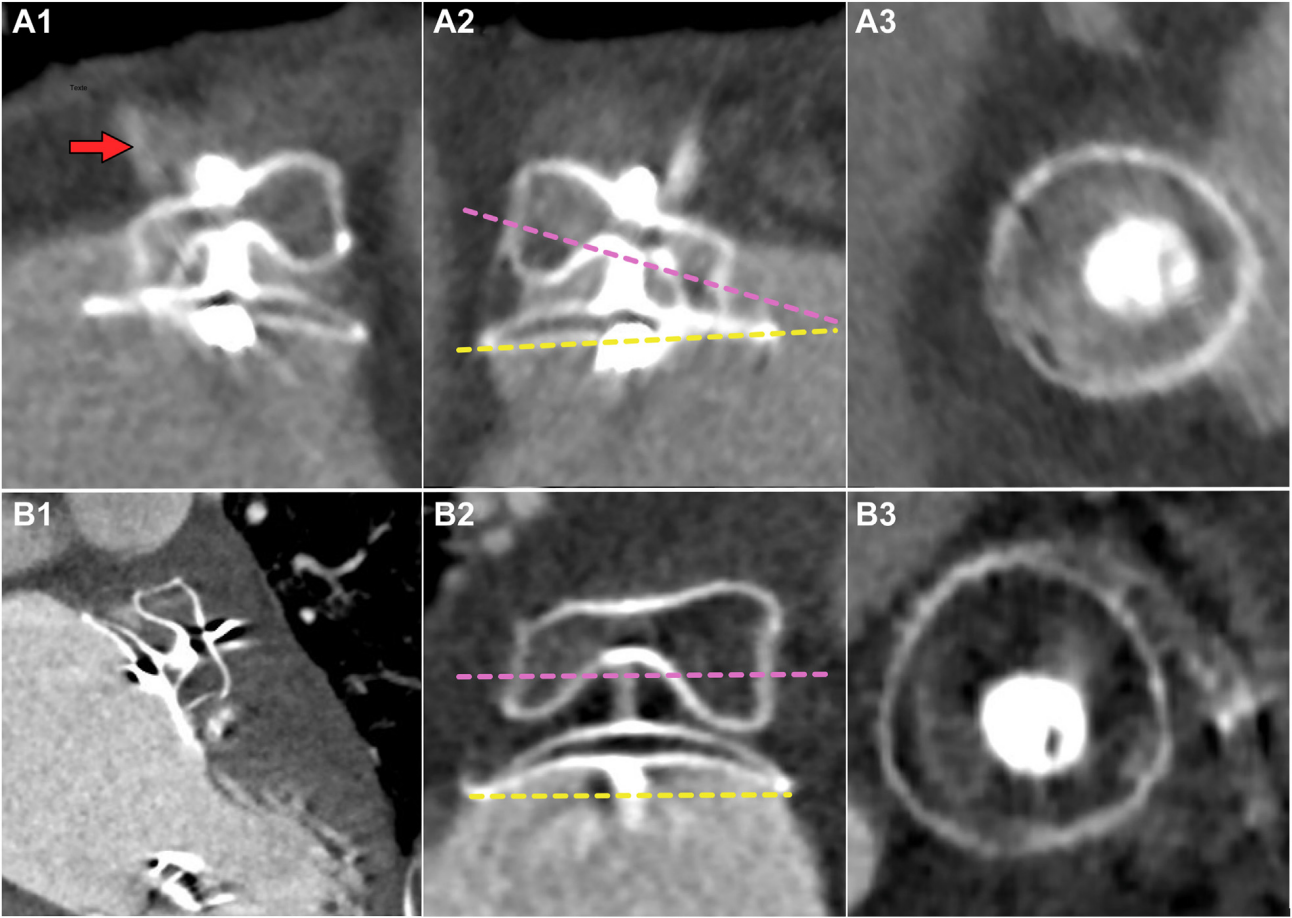
**Representative examples of patients treated with the standard (A1-A3) or steerable (B1-B3) delivery sheath.** Patient A had a cactus-shaped left atrial appendage (LAA), baseline index LA volume = 110 mL/m^2^, and was treated with 22 mm AMULET device implantation. The follow-up CT scan revealed an off-axis prosthesis (A1-A2) since the lobe was not perpendicular to the appendage wall on the landing zone. In addition, persistent LAA patency was observed (red arrow) in relation to a peridevice leak. Disc/lobe misalignment was also identified (A2) since the axes of the disc (dashed yellow) and the lobe (dashed purple line) intercepted with an angle >30°. Patient B had cactus-shaped LAA, baseline index LA volume = 160 mL/m^2,^ and was treated with 31 mm AMULET device implantation. The device's final position was optimal with the correct apposition of the lobe on the appendage wall. There was no residual LAA patency, nor peridevice leak observed. The disc and lobe were correctly aligned.

Postprocedural CT scans were generally performed between 6 and 8 weeks after LAAC in comparable hemodynamics and clinical conditions. The presence of LAA patency, potential peridevice leak (PDL), and device-related thrombus (DRT) were assessed according to previously published methods.^13^^,^^14^ Briefly, LAA was considered as patent (PA) in case the LAA density ≥100 HU or ≥25% of that of the LA; PDL were defined as the passage of contrast medium along the lobe margins for the entire length, or part of it; DRT were defined as any hypoattenuated thickening image on the atrial surface of the LAAC device. The off-axis position of the device was defined as the nonperpendicular apposition of the AMULET lobe against the wall of the LAA at the site of the LZ.^18^ Pulmonary ridge coverage was defined as the presence of the AMULET disc beyond the pulmonary ridge, as previously reported.^19^ The lobe/disc misalignment was defined as the existence of a significant discrepancy (angle >30°) between the axes parallel to the device lobe and disc.^7^ Examples of device evaluation by CT scan are provided in Figure 1.

### End points

The primary end point was procedural success according to the Munich consensus document: exclusion of the LAA, without any device-related complications, no leak >5 mm on intraprocedural color Doppler TEE and no procedure-related complications.^20^ Secondary end points included presence of disc/lobe misalignment, patent LAA, and PDL on follow-up CT scan.

### Statistical analysis

All statistical analyses were performed using SPSS 28.0 for Mac (IBM). Quantitative variables were described as median (IQR). Categorical variables were described in terms of counts and percentages. The differences between the variables were compared by the Fisher test for qualitative variables and by the 1-way ANOVA Welch’s *t* test for quantitative variables. A *P* value <.05 was considered statistically significant.

## Results

### Baseline characteristics

**Figure 2:**
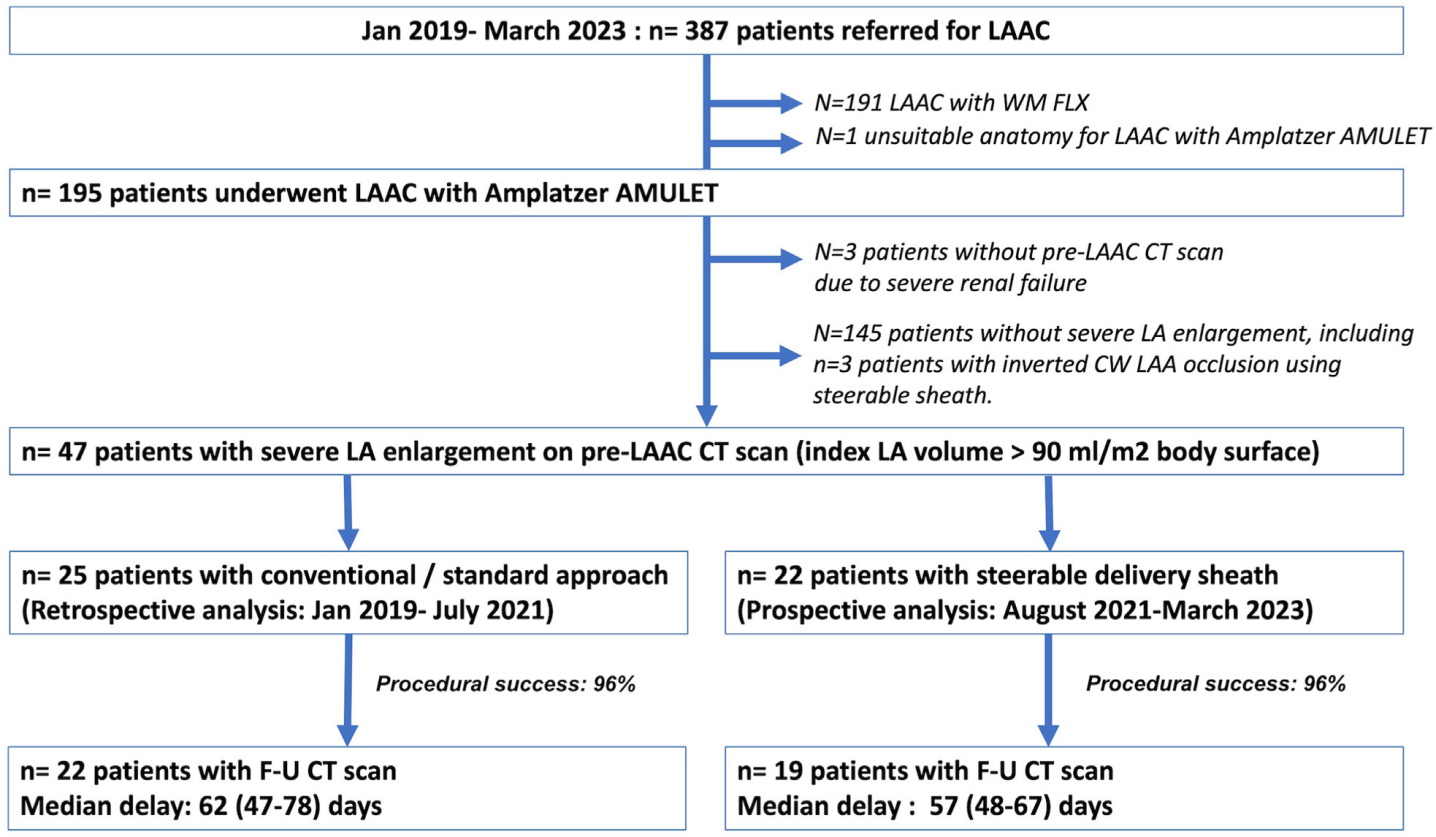
**Workflow of the study.** CT, computed tomography; LAA, left atrial appendage; LAAC, left atrial appendage closure.

The workflow of the study is provided in Figure 2. Between January 2019 and March 2023, a total of n = 387 patients underwent LAAC procedure in our institution, including n = 195 AMULET implantations. We identified a total of n = 47 patients (24%) with severely enlarged LA on baseline CT scan. SDS was used in n = 22 consecutive patients whereas n = 25 patients were included in the standard group. The baseline clinical and CT scan characteristics of the patients are given in Table 1. There were no significant differences in patient profiles, yet there was a trend toward a higher incidence of inverted chicken wing anatomy in the SDS group.

**Table 1:** Baseline characteristics.

	Standard group (n = 25)	Steerable group (n = 22)	*P*
Male sex	16 (64)	15 (68)	.76
Age, y	84 (77.8-87.0)	83.4 (76.5-86.9)	.38
CHA_2_DS_2_-VASc score	5 (4-6)	4 (4-5)	.53
Hypertension	18 (72)	19 (87)	.30
Diabetes	2 (8)	3 (14)	.65
Previous CAD	6 (24)	9 (41)	.35
Previous stroke	10 (40)	5 (23)	.21
Permanent/persistent AF	18 (72)	16 (73)	.95
LVEF, %	60 (55-62)	60 (50-63)	.59
Pre-LAAC CT scan			
LA volume, mL	207 (198-240)	205 (160-238)	.93
Index LA volume, mL/m^2^	116 (104-126)	112 (95-125)	.79
Cactus shape	9 (36)	7 (32)	.76
Chicken wing shape	8 (32)	4 (18)	.28
Inv. chicken wing shape	1 (5)	5 (23)	.09
Windsock shape	5 (20)	4 (18)	>.99
Cauliflower shape	2 (8)	2 (9)	>.99
LZ minimal diameter, mm	20.0 (17.6-23.2)	19.0 (18.0-20.5)	.15
LZ maximal diameter, mm	25.0 (21.5-28.5)	23.5 (21.0-26.5)	.15

Values are n (%) or median (IQR).

AF, atrial fibrillation; CAD, coronary artery disease; CT, computed tomography; Inv, inverted; LA, left atrium; LAAC, left atrium appendage closure; LVEF, left ventricle ejection fraction; LZ, landing zone.

### Procedural outcome

The LAAC procedure characteristics in each group are given in Table 2. The procedural success rate was comparable between SDS and standard groups (21/22 [96%] and 24/25 [96%], respectively; *P* > .99), and there was no significant difference in terms of device size. The complication rate was low, mainly driven by n = 2 cases of transient circumflex artery compression by the device before its final retrieval. In both cases, the compression disappeared after the prosthesis was reintegrated into the delivery sheath, and the procedure was completed with a smaller device. In addition, we observed 1 case of transient ischemic attack 24 hours after LAAC, but no case of air embolism nor vascular access complication was observed. The procedure duration was comparable between groups. However, when the SDS cohort was split into 2 subgroups according to the implantation data (August 2021 to September 2022 vs October 2022 to March 2023), we observed that the procedure duration was shorter in the most recent procedures (35 [30-47] vs 63 [50-92] minutes; *P* = .02) suggesting an SDS learning curve effect over time.

**Table 2:** Procedural characteristics.

	Standard group (n = 25)	Steerable group (n = 22)	*P*
Device diameter, mm	28 (25-31)	25 (25-30)	.25
Number of devices used	1 (1-1)	1 (1-2)	.1
Associated procedure			
Percutaneous mitral valve repair	0	1 (5)	.47
PFO closure	1 (4)	1 (5)	>.99
Sandwich technique	0	2 (9)	.21
Procedure duration, min	50 (38-63)	48.5 (34-69)	.66
Success rate	24 (96)	21 (96)	>.99
Complications	1 (4)	2 (9.1)	.59
Death	0	0	>.99
Pericardial effusion	0	0	>.99
Stroke/TIA	0	1 (5)	.47
Device embolization	0	0	>.99
Transient Cx artery compression	1 (4)	1 (5)	>.99
Air embolism	0	0	>.99
Vascular access complications	0	0	>.99

Values are n (%) or median (IQR).

Cx, circumflex; PFO, patent foramen ovale; TIA, transient ischemic attack.

### Follow-up CT scan data

**Figure 3:**
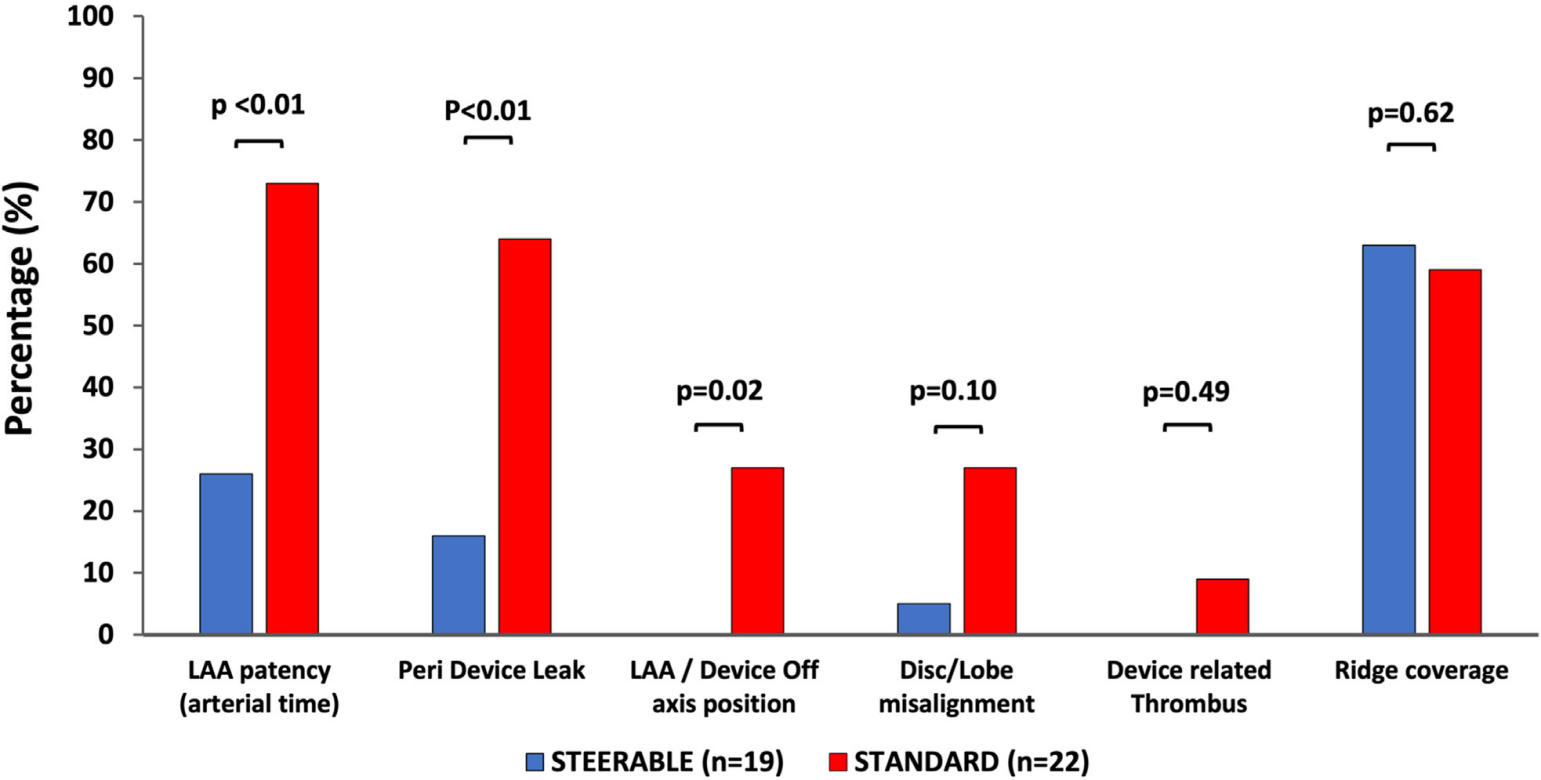
**Follow-up computed tomography scan data.** LAA, left atrial appendage.

Follow-up CT data were obtained in 91% of the patients with procedural success: n = 19 patients from the SDS and n = 22 patients from the standard group. The delay between LAAC and CT scan was comparable between groups (62 [47-78] vs 57 [48-67] days; *P* = .48). We observed a better LAA sealing in patents from the SDS group compared to the others (Figure 3) as identified by the significantly lower incidences of patent LAA on the arterial time (26% vs 72%; *P* = .005) and PDL (16% vs 64%; *P* = .004). These results were related to a better device position within the appendage, as an off-axis position was less frequently observed in the SDS group compared to the standard group (0% vs 27%; *P* = .02). In addition, we observed n = 2 DRT cases in the standard group but this incidence was not statistically significant between groups (0% vs 9%; *P* = .49). Finally, ridge coverage was also comparable between groups (63% vs 59%; *P* = .62).

## Discussion

The main results of this pilot study are as follows: (1) severe LA enlargement was a frequent finding in patients referred for LAAC; (2) the use of an SDS during LAAC with the AMULET device improved the appendage closure efficacy on imaging follow-up in these patients.

Enlarged LA is frequently observed in patients with AF. The increase of the LA volume is related to some changes in its structure (fibrosis) and function (mechanical dysfunction) that are characterized as LA remodeling, which is a common consequence of several pathophysiological mechanisms including left ventricle hypertrophy, diastolic or systolic dysfunction.^21^ New AF onset is favored by this LA remodeling and could, in turn, enhance atrium dilation because of the loss of its contractile function.^21^ This LA enlargement has been reported as a poor prognosis factor in different cardiovascular diseases. Although this condition has been infrequently investigated in patients undergoing LAAC, a previous study showed that LA volume was higher in patients with postprocedural patent LAA at CT follow-up compared to the others.^7^ Several hypotheses could explain the negative impact of this condition on the results of LAAC with double-seal occluders. First, LA enlargement creates difficulties in optimally visualizing the LAA by perprocedural TEE, which affects the procedure guidance and quality. Moreover, LA enlargement distorts angles and increases distances between the interatrial septum (site of the transseptal puncture) and LAA ostium. This condition could lead to noncoaxial device engagement and deployment within the appendage when a fixed-angle delivery sheath is used. As a consequence, off-axis device position (where the lobe is not correctly implanted within the appendage body) is more frequent and might lead to more PDL.^18^ In the current analysis, we identified severely enlarged LA in almost 1 patient out of 4. This percentage was somehow high but could be explained by the high prevalence of associated cardiac disease in our patients as well as the high percentage of persistent/permanent AF.

Steerable deflectable delivery sheaths are a possible option for overcoming these anatomical limitations and optimizing procedural outcomes. The Amplatzer SDS combines classical rotation/advancement handling movements to distal tip flexibility. Hence, SDS could adjust the orientation of the sheath tip and thus provide easier access to the appendage in case of highly angulated anatomy, posterior LAA orientation, or unsuitable/suboptimal transeptal puncture site. These conditions could be present in the most challenging cases, such as reverse chicken-wing LAA anatomies even in the absence of LA enlargement. Moreover, SDS allows a better alignment between the appendage axis and the AMULET lobe, which facilitates correct and stable device positioning (Central Illustration). Finally, the orientable tip deflection during final disc deployment helps to decrease the tension in the system and obtain a more reliable position after final release. Previous groups reported initial experience of SDS in some limited all-comers LAAC cases.^2^^,^^3^^,^^22^ However, to the best of our knowledge, the current study is the first to focus on a specific complex LAA anatomy subgroup and to provide follow-up imaging comparison with historical cohorts. Our results are in line with previously published data, showing excellent feasibility and safety profile of the system.^2^^,^^3^^,^^22^ In addition, our imaging results also suggest a better efficacy on LAA closure quality with the AMULET device, which might be related to a better and more conformable prosthesis position in the appendage. Since the most recent data suggest that persistent PDL might be associated with increased risk of long-term recurrent thrombo-embolism following LAAC,^23^^,^^24^ these results might have clinical implications for these patients.

### Limitations of the study

There was no randomization between standard sheath and SDS. The limited sample size and the missing follow-up CT scans could have affected the results. The present analysis was restricted to AMULET occluder implantation to maintain comparability between groups since there was no SDS available for the Watchman FLX system at the time of the study. Thus, we cannot exclude a potential selection bias among our patients. In addition, the translation of the results to other single-seal occluders has to be determined. Moreover, this study included patients over a long period and the results might have been positively impacted by the operators growing experience over time (learning curve effect). Finally, the Abbott SDS system used in this study was recalled by the manufacturer after the last patient enrolment because of a suspicion of increased risk of air embolism^25^ and our results have to be confirmed with other SDS devices.

## Conclusions

In this pilot study, the use of a steerable sheath for LAAC with the Amulet device in a select group of patients with severe left atrial enlargement was associated with comparable procedural success rates and better sealing of the LAA on follow-up imaging compared to procedures performed with standard sheath. This strategy might thus represent a valuable option to simplify the procedure in these patients. Whether these imaging data could be confirmed in larger populations and translate into clinical benefits remains to be determined and should be investigated in future multicenter trials.
